# Supplementary material for: Novel Driver Strength Index highlights important cancer genes in TCGA PanCanAtlas patients
Source: PeerJ. 2022 Aug 11;10:e13860. doi: 10.7717/peerj.13860 (PMC9375969; doi:10.7717/peerj.13860)
Supplement: Supplemental Information 2 [file peerj-10-13860-s002.zip › COHORTS/genes plots/detailed/2021_8_16_14_9_distribution_events_detailed_females_5.pdf]

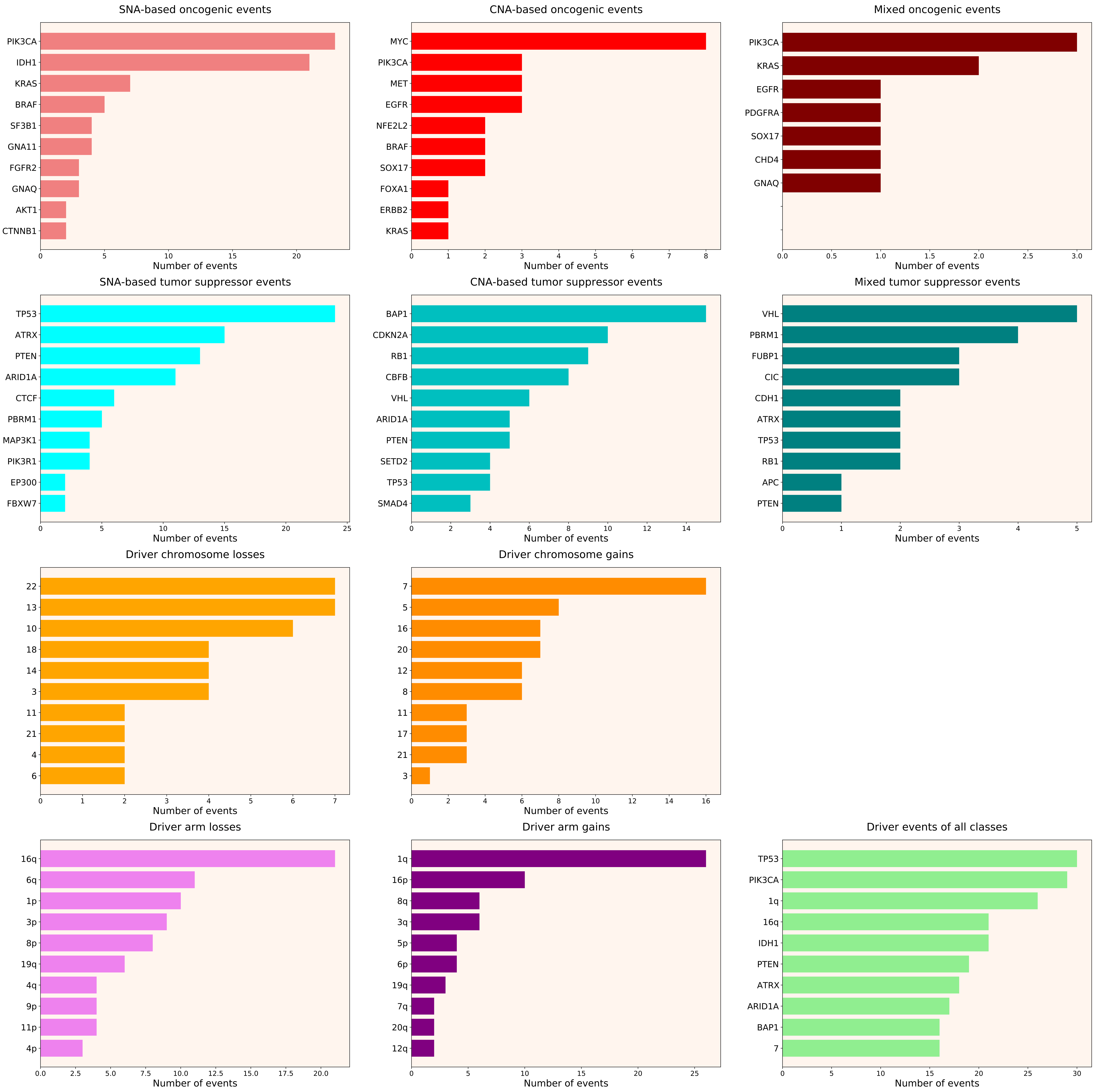

SNA-based oncogenic events
PIK3CA
IDH1
KRAS
BRAF
SF3B1
GNA11
FGFR2
GNAQ
AKT1
CTNNB1
0 5 10 15 20
Number of events
CNA-based oncogenic events
MYC
PIK3CA
MET
EGFR
NFE2L2
BRAF
SOX17
FOXA1
ERBB2
KRAS
0 1 2 3 4 5 6 7 8
Number of events
Mixed oncogenic events
PIK3CA
KRAS
EGFR
PDGFRA
SOX17
CHD4
GNAQ
0.0 0.5 1.0 1.5 2.0 2.5 3.0
Number of events
SNA-based tumor suppressor events
TP53
ATRX
PTEN
ARID1A
CTCF
PBRM1
MAP3K1
PIK3R1
EP300
FBXW7
0 5 10 15 20 25
Number of events
CNA-based tumor suppressor events
BAP1
CDKN2A
RB1
CBFB
VHL
ARID1A
PTEN
SETD2
TP53
SMAD4
0 2 4 6 8 10 12 14
Number of events
Mixed tumor suppressor events
VHL
PBRM1
FUBP1
CIC
CDH1
ATRX
TP53
RB1
APC
PTEN
0 1 2 3 4 5
Number of events
Driver chromosome losses
22
13
10
18
14
3
11
21
4
6
0 1 2 3 4 5 6 7
Number of events
Driver chromosome gains
7
5
16
20
12
8
11
17
21
3
0 2 4 6 8 10 12 14 16
Number of events
Driver arm losses
16q
6q
1p
3p
8p
19q
4q
9p
11p
4p
0.0 2.5 5.0 7.5 10.0 12.5 15.0 17.5 20.0
Number of events
Driver arm gains
1q
16p
8q
3q
5p
6p
19q
7q
20q
12q
0 5 10 15 20 25
Number of events
Driver events of all classes
TP53
PIK3CA
1q
16q
IDH1
PTEN
ATRX
ARID1A
BAP1
7
0 5 10 15 20 25 30
Number of events
